# Philadelphia Medical Schools and the U. S. Pharmacopeia

**Published:** 1908-03

**Authors:** 


### Philadelphia Medical Schools and the U. S. Pharmacopeia.

At an informal conference, called by Professor Joseph P. Remington, of the teachers named below in the medical schools of Philadelphia, the following resolution was passed:

*Resolved,* That it is of the utmost importance for accuracy in prescribing, and in the treatment of disease, that students of medicine be instructed fully as to those portions of the United States Pharmacopeia which are of value to the practitioner, and that members of the medical profession be urged to prescribe the preparations of that publication, and further that this resolution be forwarded to the medical and pharmaceutical journals, and to the teachers of medicine and therapeutics in the United States.

Signed—James Tyson, M. D.; John H. Musser, M. D.; John Marshall, M. D.; Horatio C. Wood, Jr., M. D.; H. A. Hare, M. D.; J. W. Holland, M. D.; Alfred Stengal, M. D.; David L. Edsall, M. D.; Seneca Egbert, M. D.; M. C. Thrush, M. D.; James Wilson, M. D.; E. Q. Thornton, M. D.; John V. Shoemaker, M. D.; I. Newton Snively, M. D.; J. M. Anders, M. D.; S. Colis Cohen, M. D.

February 3, 1908.

